# Correlation between CD117+ myeloma plasma cells and hematopoietic progenitor cells in different categories of patients

**DOI:** 10.1186/s12979-015-0032-1

**Published:** 2015-06-04

**Authors:** Fanny Pojero, Alessandra Casuccio, Francesco Di Bassiano, Francesco Gervasi, Giuseppina Colonna Romano, Calogero Caruso

**Affiliations:** Dipartimento di Biopatologia e Biotecnologie Mediche, Universita’ degli Studi di Palermo, Corso Tukory 211, 90134 Palermo, Italy; Dipartimento dei Servizi, U.O.S.D. Laboratorio Specialistico Oncologia, Ematologia e Colture Cellulari per Uso Clinico, ARNAS Civico, Piazza Nicola Leotta 4, 90127 Palermo, Italy; Dipartimento di Scienze per la Promozione della Salute e Materno Infantile “G. D’Alessandro”, Universita’ degli Studi di Palermo, Via del Vespro 133, 90133 Palermo, Italy; U.O.C. di Oncoematologia, ARNAS Civico, Piazza Nicola Leotta 4, 90127 Palermo, Italy

**Keywords:** Multiple myeloma, MGUS, CD117, Hematopoietic progenitor cell, Flow cytometry

## Abstract

**Background:**

Multiple myeloma (MM) is a neoplastic disorder of plasma cells interesting mainly the elderly. MM remains an incurable disease, mostly because of the strong interplay between clonal plasma cells (cPCs) and bone marrow (BM) microenvironment. Multiparameter flow cytometry (MFC) allows the simultaneous study of the cPC immunophenotype and alterations involving other cells in BM, but rarely these data are interpreted as connected. One exception to this habit are previous studies about relationship between CD117 cPC positivity and hematopoietic progenitor cell (HPC) distribution in newly diagnosed patients. Thus we were interested in verifying the distribution of BM CD34+ HPCs in healthy controls, and monoclonal gammopathy of undetermined significance (MGUS) patients and various categories of responding/relapsing MM subjects divided according to CD117 positivity.

**Results:**

Our data completely agree with precedent reports as regards untreated patients. In the group with progression of disease, CD117- patients exhibited a lower CD34 + CD19-/CD34 + CD19+ ratio vs CD117+ subjects. Among CD117- cases, newly diagnosed patients exhibited differences in distribution of HPCs vs responding myeloma subjects and patients with progressive disease. These differences reached statistical significance comparing CD117- newly diagnosed with CD117- responding cases, as reflected by CD34 + CD19-/CD34 + CD19+ ratio. In turn, no differences emerged comparing CD117+ treated and untreated patients.

**Conclusions:**

We demonstrate that administration of treatment and depth of reached response/presence of relapse imply a distinct regulation in distribution of CD34+ HPC subsets in CD117- and CD117+ patients. These differences become evident comparing untreated and treated CD117- patients, but they are impossible to detect in CD117+ cases.

**Electronic supplementary material:**

The online version of this article (doi:10.1186/s12979-015-0032-1) contains supplementary material, which is available to authorized users.

## Background

Multiple myeloma (MM) is an incurable neoplastic plasma cell disorder characterized by proliferation of clonal/aberrant malignant plasma cells (cPCs) in bone marrow, and presence of monoclonal immunoglobulin (M-protein) in serum and/or urine, associated with immunodeficiency and related organ or tissue impairment [1–3]. With a median age at diagnosis of 65–70 years [3], MM is a disease affecting mainly elderly subjects. MM is usually preceded by a premalignant PC proliferative stage characterized by asymptomatic M-protein production known as monoclonal gammopathy of undetermined significance (MGUS), which is associated with a rate of progression to multiple myeloma of 1 % per year [4, 5]. In MM long term control of the disease is still an elusive objective. Despite the dramatic progress in therapeutic approaches, due to the introduction of novel categories of drugs (proteasome inhibitors and immunomodulatory agents) [6], no curative strategies have currently been defined. Even patients undergoing to high-dose therapy (HDT) and autologous stem cell transplantation (ASCT) may experiment relapse [7, 8]. This phenomenon is strictly related to the strong interplay between cPCs and bone marrow (BM) microenvironment. Residual cPCs may escape therapeutic effects in bone marrow niches, which have been proved to be able to enhance cPC survival and modulate immune system ability to eradicate malignant cells [3, 9, 10]. It has now become clear that, in order to cure MM, targeting BM players other than MM cells, and identifying the role of BM microenvironment in response to therapeutic intervention are necessary. In diagnosis and managing of MM and its preceding condition MGUS, multiparameter flow cytometry (MFC) plays a key role, allowing enumeration of cPCs, and definition of their immunophenotypic characteristics in comparison with normal/reactive polyclonal plasma cells (nPCs). Although MFC makes it possible to study simultaneously the immunophenotype of cPCs and the alterations involving other cellular components in the BM microenvironment in the same samples (belonging or not to the immune system) [11–16], rarely these data are used to make a connection between immunophenotypic PC characteristics and modifications in BM populations. This is true also in studies regarding hematopoietic progenitor cell subset (HPC) distribution, which has been shown to be impaired in MM patients at diagnosis and relapse [14–16]. CD117 may be aberrantly expressed on cPCs in MM and MGUS [11, 12, 17, 18], and positivity for this marker confers a favourable prognosis [12, 17, 18]. Schmidt-Hieber et al. [19] hypothesized that CD117 might act as an anchor, favouring the adhesion of cPCs to myeloid precursor-associated BM niches, with this interaction being mediated by c-kit ligand expressed by stromal cells [19]; no clear mechanism has been defined yet. Previous studies demonstrated that in newly diagnosed patients, no differences were observed as regards CD34 + CD38-/dim fraction, but the ratio between BM CD34 + CD19-CD38+ and CD34 + CD19 + CD38+ progenitors was increased in CD117- patients compared to CD117+ subjects [19]. Despite the recent augmented interest in hematopoietic progenitor cell (HPC) distribution, depending on the debate about the utility of quantification of HPC fractions in grafts [20–22], observations about CD34 + CD19- and CD34 + CD19+ subsets and related ratio (CD34 + CD19-/CD34 + CD19+ ratio) were not examined in responding and relapsing patients, and currently it is not known whether differences in CD34 + CD19- and CD34 + CD19+ (Pro-B) cell fractions are preserved in treated patients or may influence depth of response. In this study, we verified distribution of BM CD34+ HPCs in healthy controls, MGUS patients and various categories of responding/relapsing MM subjects. Moreover, after dividing patients accordingly to CD117 expression or absence on PC surface, we compared differences in percentage of CD34 + CD19- HPCs and Pro-B cells to detect a potential mechanism related to influence of CD117 positivity on prognosis.

## Results

### Characteristics of patients and plasma cell analysis

A total of 63 subjects (39 male and 24 female, no intergroup significant differences) was included in this study. Patients in Progressive group were significantly older (75.13 ± 10.06 years) than Complete (61.1 ± 8.85 years, *p* = 0.029) and Therapy (61.21 ± 9.01 years, *p* = 0.017) subjects, while no significant differences emerged with Control (62 ± 5.36 years), MGUS (70.8 ± 7.3 years) and New (63.58 ± 12.8). Autologous stem cell transplantation was performed in 6 Complete, 6 Therapy and 2 Progressive patients at least 12 months before the time of this study (not significant). As regards therapeutic regimen no statistically significant differences were detected. Bortezomib + Dexamethasone based treatment was administered to 5 Therapy and 3 Progressive patients. Thalidomide as monotherapy or in combination with bortezomib was given to 1 Complete and 3 Therapy patients respectively. Lenalidomide or lenalidomide containing regimens were the treatment of choice in 4 Complete and 1 Progressive subjects, while Pomalidomide was administered to 1 Progressive patient. Finally, a total of 13 patients (5 Complete, 6 Therapy and 2 Progressive) underwent suspension of therapy for at least 15 days before this study was perfomed. No differences emerged for ISS staging at diagnosis (ISS I, 3 Complete, 3 Therapy and 3 New; ISS II 7 Complete, 4 Therapy, 5 New and 5 Progressive; ISS III 7 Therapy, 4 New and 3 Progressive). Instead as regards Durie-Salmon staging at diagnosis, the cases were distributed as follows: IA, 2 Complete, 4 New and 4 Progressive; IIA, 1 Complete, 5 Therapy, 5 New and 2 Progressive; IIB, 3 Therapy, 2 New and 2 Progressive; IIIA, 6 Complete and 2 Therapy (Complete vs New, *p* = 0.018); IIIB, 1 Complete, 4 Therapy and 1 New.

According to literature [18], nPCs were always CD117-. In turn, cPCs were CD117+ in 50 % (5) of MGUS, 42.86 % (6) of Therapy, 59.17 (7) of New and 62.5 % (5) of Progressive patients (not significant). Considering each group separately, characteristics of patients were homogeneous comparing CD117- and CD117+ cases, except in group New, in which none of CD117+ patients presented characteristics of ISS III stage (vs 4/5 of CD117- patients, *p* = 0.01), and in group Progressive, in which CD117+ patients were significantly older than CD117- ones (81.20 ± 4.09 vs 65 ± 8.66 years respectively, *p* = 0.01).

**Fig. 1:**
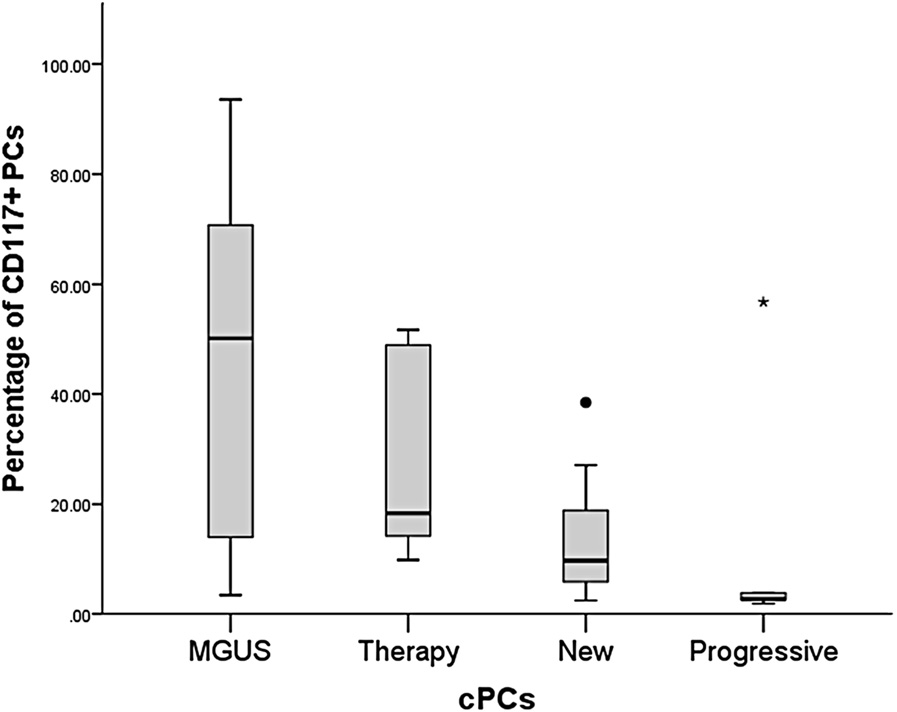
Percentages of CD34 + cells (A), CD34+CD19- (B) and CD34+CD19 + (C) fractions, and CD34 + CD19-/CD34 + CD19-ratios (D) for all groups. Box plots indicate the median and 25th and 75th percentiles. Whiskers indicate minimum and maximum values. •,* = outliers. * indicate values that are more distant than 1.5 interquartile ranges considering the nearest edge of the represented box; • indicate values that are more distant than 3 interquartile ranges considering the nearest edge of the represented box, as reported in Ref. [31]. The Kruskal-Wallis statistic test with pairwise comparisons was used for this analysis; p values of pairwise comparisons are indicated

Significant differences were detected comparing median percentage of total PCs in New [8.64 % (0.26–60.50 %)] and Progressive [6.47 % (0.75–30.29 %)] with Control [0.15 % (0.01–1.71 %), vs New *p* < 0.0005, vs Progressive *p* = 0.001], Complete [0.38 % (0.03–2.49 %), vs New *p* = 0.006, vs Progressive *p* = 0.013] and Therapy [0.48 % (0.12–14.40 %), vs New *p* = 0.012 and vs Progressive *p* = 0.029], while no difference was observed with MGUS [1.39 % (0.36–2.40 %)]. All PCs in Control and Complete were polyclonal, whereas nPCs were 19.12 % (5.24–82.52 %) in MGUS, 29.24 % (0.14–96.12 %) in Therapy, 1.18 % (0.05–66.12 %) in New (vs Therapy *p* = 0.016), and 1.60 % (0.25–12.88 %) in Progressive. As regards cPCs, New showed the most elevated median percentage [98.83 % (33.88–99.95 %)] compared with Therapy [70.76 % (3.88–99.86 %), *p* = 0.016], but no difference emerged in comparisons with MGUS [80.88 % (17.48–94.76 %)] and Progressive [98.40 % (87.12–99.75 %)]. As regards proportion of CD117+ cPCs, we observed a trend depicting the highest percentage of CD117+ plasma cells in MGUS and the lowest in Progressive (Fig. 1).

Intragroup comparisons between CD117+ and CD117- cases as regards percentages of total plasma cells, nPCs and cPCs are indicated in Table 1. In New, percentage of nPCs was significantly higher and percentage of cPCs was significantly lower in CD117+ cases vs CD117- ones. The same picture was recorded in MGUS and Progressive groups comparing CD117+ with CD117- patients, although it did not reach statistical significance. Instead, in Therapy group, we observed an extremely wide range of percentages for both nPCs and cPCs in CD117- cases in comparison with CD117+ patients.

**Table 1:** Intragroup comparisons between CD117+ and CD117- cases for plasma cells

Group		CD117+ cases	CD117- cases	*p* value
MGUS	Tot PCs	1.30	1.48	NS
		(0.71–2.40)	(0.36–2.08)	
	nPCs	19.17	10.74	NS
		(6.17–82.52)	(5.24–51.64)	
	cPCs	80.83	89.26	NS
		(17.48–93.83)	(48.36–94.76)	
Therapy	Tot PCs	0.62	0.41	NS
		(0.14–14.40)	(0.12–1.15)	
	nPCs	25.19	71.79	NS
		(0.14–70.80)	(1.23–96.12)	
	cPCs	74.82	28.21	NS
		(29.20–99.86)	(3.88–98.77)	
New	Tot PCs	6.48	13.03	NS
		(0.26–20.19)	(0.44–60.50)	
	nPCs	1.68	0.32	0.01
		(0.75–66.12)	(0.05–1.32)	
	cPCs	98.32	99.68	0.01
		(33.88–99.25)	(98.68–99.95)	
Progressive	Tot PCs	2.14	9.03	NS
		(0.75–30.29)	(8.11–23.98)	
	nPCs	6.38	0.32	NS
		(0.59–12.88)	(0.25–1.67)	
	cPCs	93.62	99.68	NS
		(87.12–99.41)	(98.33–99.75)	

Results are expressed as median percentage (range)

*Tot PCs* total plasma cells, *nPCs* normal/reactive polyclonal plasma cells (out of total plasma cells), *cPCs* clonal/aberrant plasma cells (out of total plasma cells), *NS* not significant

The Mann-Whitney *U*-test was used for this analysis

### Relationship between CD117 positive and negative cPCs and CD34+ bone marrow HPCs

**Fig. 2:**
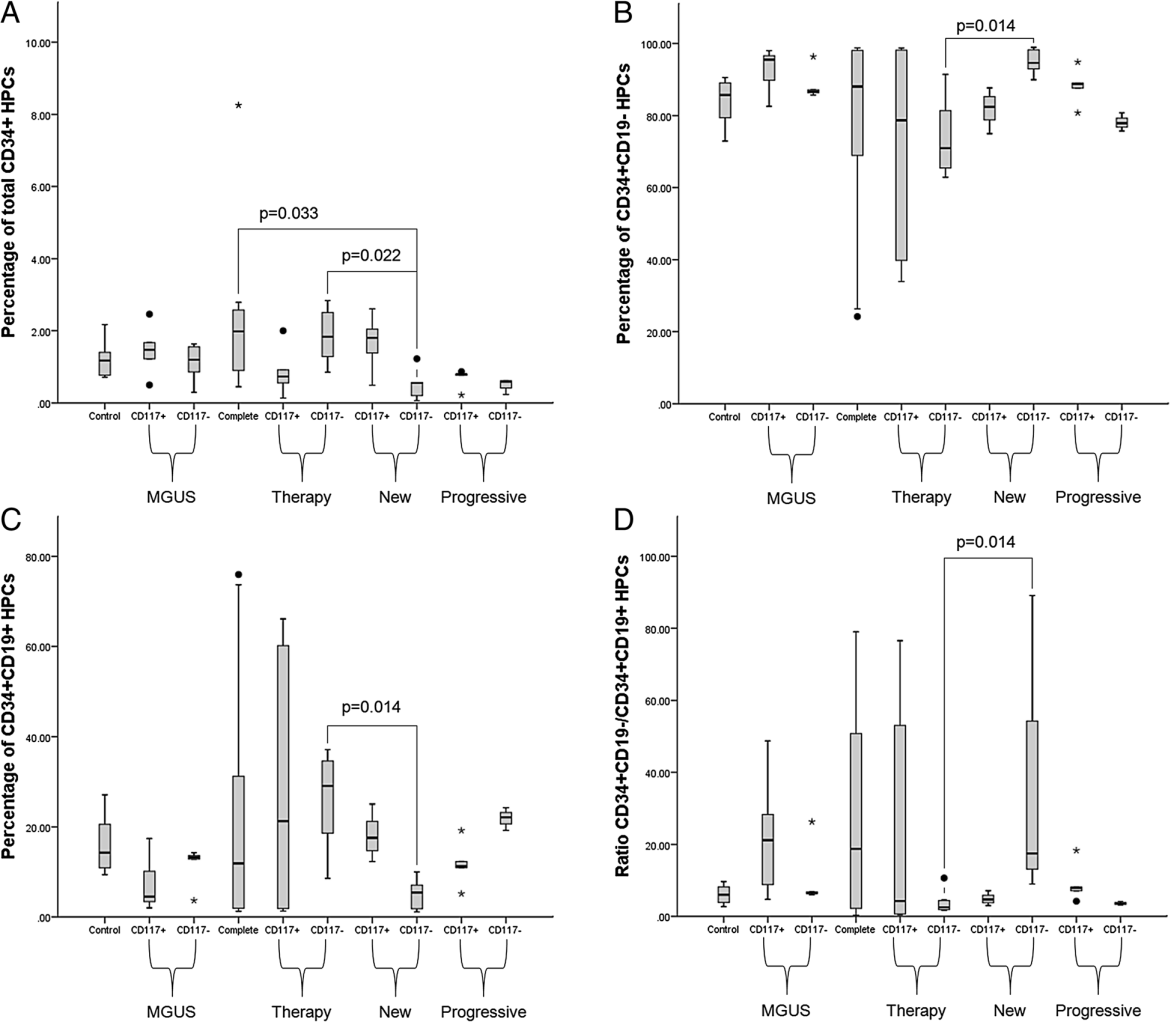
Percentages of CD34+ cells (**a**), CD34+CD19- (**b**) and CD34+CD19+ (**c**) fractions, and CD34 + CD19-/CD34 + CD19- ratios (**d**) for all groups. Box plots indicate the median and 25th and 75th percentiles. Whiskers indicate minimum and maximum values. •,* = outliers. * indicate values that are more distant than 1.5 interquartile ranges considering the nearest edge of the represented box; • indicate values that are more distant than 3 interquartile ranges considering the nearest edge of the represented box, as reported in Ref. [31]. The Kruskal-Wallis statistic test with pairwise comparisons was used for this analysis; p values of pairwise comparisons are indicated

No statistical significant differences emerged comparing percentage of total CD34+ HPCs, CD34 + CD19+ cells (out of total CD34+ HPCs) and CD34 + CD19- cells (out of total CD34+ cells), as well as CD34 + CD19-/CD34 + CD19+ ratio, despite the fact that Therapy exhibited the highest CD34 + CD19+ fraction and the lowest CD34 + CD19- fraction and CD34 + CD19-/CD34 + CD19+ ratio (Table 2). Performing intragroup comparisons between CD117+ vs CD117- cases, we noticed that Therapy CD117- patients showed a higher percentage of total CD34+ cells compared to Therapy CD117+ patients. In New, CD117+ subjects exhibited a higher percentage of total CD34+ and CD34 + CD19+ cells vs CD117- patients; in turn, CD117- cases showed a more extended proportion of CD34 + CD19- cells and a higher CD34 + CD19-/CD34 + CD19+ ratio. The frame seemed to be reversed in Progressive group: CD117+ patients exhibited higher median fraction of CD34 + CD19- cells, and CD117- presented a higher median CD34 + CD19+ percentage and a lower CD34 + CD19-/CD34 + CD19+ ratio (Table 3). Similarly in MGUS and Therapy, median percentage of CD34 + CD19- progenitors was larger and median percentage of CD34 + CD19+ cells was reduced comparing CD117- cases vs CD117+ ones (Table 3, not statistically significant). Exploring relationship between percentage of CD117+ cPCs and CD34 + CD19-/CD34 + CD19+ ratio, Spearman’s correlation coefficients were −0.718 (*p* = 0.009) for New, and 0.952 (*p* < 0.0005) for Progressive. To further deepen how CD117 positivity or negativity may influence distribution of HPCs, we compared Control and Complete data with results obtained from patients in all groups divided according to presence or absence of CD117 on cPC surface. When we compared results from Control, Complete and CD117- cases (Fig. 2a–d), we noticed that CD117- New cases showed the lowest percentage of total CD34+ BM cells compared to Complete and CD117- Therapy patients. In turn, percentage of CD34 + CD19- cells was higher in CD117- New vs CD117- Therapy (*p* = 0.014) and Progressive (not significant) subjects (Fig. 2b), while inverted situation was recorded for CD34 + CD19+ cells, with CD117- New cases depicting a reduced percentage of these progenitors in comparison with Therapy (*p* = 0.014) and Progressive (not significant) patients (Fig. 2c). As expected, CD34 + CD19-/CD34 + CD19+ ratio was increased in CD117- New subjects vs Therapy and Progressive (Fig. 2d). When we compared Control, Complete and CD117+ cases, no statistical significant differences were observed (Fig. 2a–d).

**Table 2:** Percentages of total CD34+ cells and CD34+ fractions

	Tot CD34+	CD34 + CD19-	CD34 + CD19+	Ratio
Control	1.17	85.74	14.26	6.01
	(0.72–2.17)	(72.94–90.62)	(9.38–27.06)	(2.70–9.66)
MGUS	1.34	88.51	11.49	7.82
	(0.30–2.46)	(82.58–97.99)	(2.01–17.42)	(4.74–48.75)
Complete	1.98	88.10	11.91	18.75
	(0.45–8.26)	(24.18–98.75)	(1.25–75.97)	(0.32–79)
Therapy	1.28	73.12	26.88	2.73
	(0.14–2.84)	(33.91–98.71)	(1.29–66.09)	(0.51–76.52)
New	1.26	87.11	12.90	6.77
	(0.06–2.61)	(74.98–98.89)	(1.11–25.02)	(3–89.09)
Progressive	0.70	84.24	15.76	5.66
	(0.22–0.87)	(75.73–94.83)	(5.17–24.27)	(3.12–18.34)

Results are presented as median values (range)

Tot CD34+, total CD34+ hematopoietic progenitor cells; CD34 + CD19-, fraction of CD34 + CD19- cells (out of total CD34+ cells); CD34 + CD19+, fraction of CD34 + CD19+ cells (out of total CD34+ cells); Ratio, CD34 + CD19-/CD34 + CD19+ cellular fraction ratio

The Kruskal-Wallis statistic test with pairwise comparisons was used for this analysis (no significant results)

**Table 3:** Intragroup comparisons between CD117+ and CD117- cases for total CD34+ cells and CD34+ fractions

Group		CD117+	CD117-	*p* value
MGUS	Tot CD34+	1.47	1.19	NS
		(0.50–2.46)	(0.30–1.63)	
	CD34 + CD19-	95.49	86.72	NS
		(82.58–97.99)	(85.73–96.34)	
	CD34 + CD19+	4.51	13.28	NS
		(2.01–17.42)	(3.66–14.27)	
	Ratio	21.17	6.53	NS
		(4.74–48.75)	(6.01–26.32)	
Therapy	Tot CD34+	0.73	1.83	0.013
		(0.14–2)	(0.85–2.84)	
	CD34 + CD19-	78.71	70.97	NS
		(33.91–98.71)	(62.86–91.42)	
	CD34 + CD19+	21.29	29.04	NS
		(1.29–66.09)	(8.58–37.14)	
	Ratio	4.27	2.50	NS
		(0.51–76.52)	(1.69–10.66)	
New	Tot CD34+	1.80	0.56	0.018
		(0.49–2.61)	(0.06–1.22)	
	CD34 + CD19-	82.42	94.59	0.003
		(74.98–87.70)	(90–98.89)	
	CD34 + CD19+	17.58	5.41	0.003
		(12.30–25.02)	(1.11–10)	
	Ratio	4.69	17.48	0.003
		(3–7.13)	(9–89.09)	
Progressive	Tot CD34+	0.80	0.60	NS
		(0.22–0.87)	(0.23–0.62)	
	CD34 + CD19-	88.75	77.88	0.036
		(80.79–94.83)	(75.73–80.77)	
	CD34 + CD19+	11.25	22.12	0.036
		(5.17–19.21)	(19.23–24.27)	
	Ratio	7.89	3.52	0.036
		(4.21–18.34)	(3.12–4.20)	

Results are expressed as median percentage (range)

Tot CD34+, total CD34+ hematopoietic progenitor cells; CD34 + CD19-, fraction of CD34 + CD19- cells (out of total CD34+ cells); CD34 + CD19+, fraction of CD34 + CD19+ cells (out of total CD34+ cells); Ratio, CD34 + CD19-/CD34 + CD19+ cellular fraction ratio

The Mann-Whitney *U*-test was used for this analysis

## Discussion

The interaction of cPCs with BM microenvironment is fundamental to ensure development and progression of MM. Destruction of BM homeostasis, by a complex system of direct, autocrine and paracrine interactions between components of the BM microenvironment and cPCs, influences proliferation and triggering of anti-apoptotic mechanisms [3, 10]. Normal hematopoiesis is impaired in MM subjects, with anemia being one of MM characteristic clinical features [8]. Precedent studies demonstrated alterations in BM distribution of HPCs in MM subjects. Compared to healthy donors, a substantial reduction of CD34+ HPC subsets and CD19 + CD38 + CD34+ Pro-B cells in terms of absolute cell count and proportion of mononuclear cells respectively was described in untreated MM subjects [15]. Similarly, percentage of total CD34+ cells and CD34 + CD19+ cells (both defined as proportion of total leukocytes excluding PCs) was proven to be decreased at presentation and relapse, but not in patients at plateau/remission, vs normal individuals [14]. Coherently, a more recent report confirmed reduction of percentage of CD34+ HPCs (from whole BM cellularity) and CD34 + CD38 + CD19+ progenitors (out of total CD34+ HPCs) in BM of MM patients vs healthy controls [16]. In all listed studies, no categorization of patients on the basis cPC immunophenotype was performed. In newly diagnosed patients, divided accordingly to CD117 positivity and negativity, the percentage of CD34 + CD19 + CD38+ progenitor cells was reported to be higher in CD117+ vs CD117- cases, leading to a significant decrease in CD34 + CD19-/CD34 + CD19+ ratio [19]. On the basis of precedent reports, we were interested in exploring the relationship between presence/absence of CD117 on cPCs surface and distribution of HPCs into CD34 + CD19- and Pro-B subsets in MGUS, newly diagnosed, stringent complete responders, treated (but not complete responder) patients, and subjects with progressive disease. We did not observed significant differences in frequency of CD117 positivity comparing all categories of patients, contradicting previous reports [18]. However this discrepancy could be related to variations in sample size. In New, only CD117- were classified as stage ISS III, while none of CD117+ cases showed association with the most advanced ISS stage. These data, together with the trend exhibited by CD117+ cPCs (the highest percentages recorded in MGUS, decreasing through Therapy and New, to the most reduced fraction observed in Progressive) strongly recalls the association of CD117 negativity with features of a more aggressive disease in MM [17], and supports the hypothesis that CD117+ clones might be deleted during progression [18]. In intragroup comparisons of percentages of total, normal and clonal PCs, we observed that in New CD117+ patients nPCs were significantly higher and cPCs were significantly lower vs New CD117- subjects, accordingly to precedent reports [19]. No significant differences were observed in other groups, but the exact mechanism ruling expansion of cPCs vs nPCs remains to be elucidated. As regards distribution of CD34+ HPCs, we did not recorded general variations in percentage of CD34+ HPCs, and CD34 + CD19- and Pro-B cell subsets comparing all groups among them. This is openly conflicting with precedent papers [14–16], but may be easily explained considering three important factors: 1) the studies were conducted following different methods of measuring and expressing fractions of CD34+ HPCs; 2) the statistical analysis was performed through different tests (we needed to use specific tests for multiple comparisons); 3) patients were not divided in groups according to the immunophenotype of cPCs, so we cannot evaluate carefully the impact of CD117 positivity on previously reported data. In fact, carrying on our analysis, we noticed that CD117 has a strong influence on CD34+ HPC distribution both in New and Progressive groups, but with different outcomes. In New our findings are consistent with those described by Schmidt-Hieber et al. [19], with a significant inverse correlation between CD117 positivity and CD34 + CD19-/CD34 + CD19+ ratio. Instead, the opposite situation was recorded in Progressive, with a significant direct correlation between CD117 and CD34 + CD19-/CD34 + CD19+ ratio, reflected by detected measured fractions of CD34 + CD19- and Pro-B cells. Interestingly, also in Therapy and MGUS groups we evidenced that CD117 positivity is associated with a reduction in median percentage of CD34 + CD19+ HPCs and an increase in median percentage of CD34 + CD19- cells, although this results did not reach statistical significance. A previously elaborated hypothesis underlined the possibility that CD117+ cPCs may consume stem cell factor and occupy BM stem cell niches, thus perturbing normal hematopoietic process [19]. Moreover, the same authors proposed that in CD117+ patients, nPCs may exert a more pronounced homeostatic role in comparison with CD117- subject; this property of nPCs may be a function of the adhesive action of CD117+ on cPCs, which may prevent cPCs to invade B cell precursor niches [19]. Our data suggest that in MGUS and Therapy patients the presence of an extended amount of nPCs may counteract the effect on HPCs mediated by expansion of CD117+ cPCs, whereas in Progressive patients the percentage of CD117+ cPCs is so small (Fig. 1) that it cannot exert the same modulation on HPCs observed in New subjects. However, given the small sample size, confirmation by a larger cohort of patients is recommendable. The most surprising results emerged from analysis of Control and Complete data with measured values for CD117- cases. Total CD34+ HPCs were less expanded in CD117- New vs both Complete and CD117- Therapy subjects, thus reflecting a possible reorganization of BM niches in patients able to respond to treatment. Moreover, significant differences were observed between CD117- New and Therapy patients, with New displaying a more extended CD34 + CD19- fraction and a reduced Pro-B population compared to Therapy. This was concretized in a higher CD34 + CD19-/CD34 + CD19+ ratio in CD117- New cases vs CD117- Therapy patients. Similar differences in CD34 + CD19- and CD34 + CD19+ HPC distribution emerged comparing CD117- New with CD117- Progressive patients, but statistical significance was not achieved. In turn, when we compared Control, Complete and CD117+ subjects, no significant variations in percentage of CD34 + CD19- and CD34 + CD19+ cells emerged. These pieces of information are extremely intriguing, since they clearly demonstrate administration of treatment and depth of reached response/presence of relapse imply a distinct regulation in distribution of CD34+ HPC subsets in CD117- and CD117+ patients. Such a alteration in BM stem cell niche composition clearly rises comparing untreated and treated CD117- patients, but it is impossible to detect in CD117+ cases. Given that significant alterations in distribution of CD34+ HPC subsets exclusively regards patients unable to reach complete response, it will be interesting to evaluate the influence of other immunophenotypes (not described in this studies) on the ability of CD117- patients to achieve a deeper response. In addition, a possible future step might be the study of an eventual correlation between plasma cell immunophenotypic characteristics and mobilization/graft contents.

## Conclusions

In conclusion, we confirmed previous trends in CD34+ HPC subset distribution in newly diagnosed patients divided according to CD117 positivity. Moreover, we provided some insights in CD34+ HPC distribution in relapsing patients. We also describe different impact of treatment on CD34+ HPCs in CD117- patients vs CD117+ subjects, thus opening the debate about effect of CD117 on mechanism determining prognosis.

## Methods

### Patients and BM samples

Control specimens consisted of 9 BM samples from patients who were suspected to have a haematological disease and revealed to be non onco-hematological subjects (group Control). These patients have no history of MM, MGUS or lymphoid/myeloid neoplasm. BM samples of 44 patients with MM and 10 patients with MGUS submitted to our laboratory for routine analysis were evaluated by MFC. For every patient clinical chemical and immunological profiles, as well as reference intervals were provided by the U.O. Patologia Clinica - Laboratorio Analisi Cliniche of ARNAS Civico, Palermo (Italy). Disease stage was defined according to Durie-Salmon and ISS staging criteria [23, 24]. Response to therapy was defined conforming to Bird et al. [25]. Of MM samples, 12 were obtained at presentation (group New), 8 from patients with progressive disease (group Progressive), 14 from patients unable to reach complete response (4 Very Good Partial Response, 6 Partial Response and 4 Stable Disease - group Therapy) and 10 from patients who achieved stringent CR (group Complete). MGUS patients were considered as a separate group (group MGUS). Clinical data and history for MGUS and MM cases were provided by U.O. Oncoematologia of ARNAS Civico, Palermo (Italy). Informed consent procedures and forms were proposed to and approved by the ARNAS Civico Medical Ethics Committee. Written informed consent was given by all subjects in line with the Declaration of Helsinki. BM samples were collected in EDTA tubes and processed in one hour since collection.

### Multiparameter flow cytometry

Details about antibodies and instrument are indicated in Tables S1 and S2 respectively [see Additional file 1]. Specimens were fragmented with a sterile syringe and filtered using a 80 μm filter; nucleated cells were enumerated using UniCel® DxH™ 800 Coulter® Cellular Analysis System (Beckman Coulter, Miami, FL, USA) and brought to a final concentration of 10^6^cells/100 μl with PBS w/o calcium and magnesium (EuroClone, Milan, Italy). To stain surface and intracellular markers, the following combinations of antibodies were used: Tube 1, Cytκ_FITC_/Cytλ_PE_/CD38_PC5.5_/CD56_PC7_/CD138_APC_/CD27_APC-AlexaFluor 750_/CD19_PB_/CD45_KO_; Tube 2, CD27_FITC_^#^/CD56_PE_/CD38_PC5.5_/CD117_PC7_/CD138_APC_/CD34_APC-AlexaFluor 750_/CD19_PB_/CD45_KO_ (Cyt, Cytoplasmic; FITC, Fluorescein Isothiocyanate; PE, R-Phycoerythrin; PC5.5, R-Phycoerythrin-Cyanin 5.5; PC7, R-Phycoerythrin-Cyanin 7; APC, Allophycocyanin; PB, Pacific Blue; KO, Krome Orange). All antibodies were purchased from Beckman Coulter (Miami, FL, USA), except for # which was purchased from BD Biosciences (San Jose, CA, USA). For staining of surface markers, 100 μl of each sample were incubated with the opportune combinations of antibodies for 15 min in the dark. Erythrocytes were lysed adding 1 ml of VersaLyse™ Lysing Solution and incubating tubes for 20 min in the dark. For intracellular staining of kappa and lambda light chains, 50 μl of sample were washed 5 times with 2 ml of PBS w/o calcium and magnesium (EuroClone, Milan, Italy), and processed with PerFix-nc (Beckman Coulter, Miami, FL, USA) following instructions. Samples were all acquired with Navios™ Flow Cytometry System, data were collected with Navios v1.0 Software (Beckman Coulter, Miami, FL) and then analyzed with Kaluza® Flow Cytometry Analysis Software v1.3 (Beckman Coulter, Miami, FL, USA). Daily testing of instrument was performed as indicated: standardization of light scatter, fluorescence intensity and optimal hydrodynamic focusing instrument settings were verified using Flow-Set Pro Fluorospheres (Beckman Coulter, Miami, FL, USA); compensation matrix for each combination of antibodies was tested with CYTO-COMP™ Cell Kit (Beckman Coulter, Miami, FL, USA); optical alignment and fluidics were checked using Flow-Check Pro Fluorospheres (Beckman Coulter, Miami, FL, USA). In order to identify PCs, a combination of CD38, CD138 and CD45 together with light scatter properties was used; the first gate was set on CD38 vs CD138 as suggested [11]. Distinction between normal/reactive and clonal plasma cell compartments was performed basing on their most frequent aberrant phenotypes (CD38, CD19, CD27, CD117, CD56 and CD45); results were confirmed by the presence of clonal restriction in population showing the abnormal phenotype, and the absence of restriction in normal PCs [11, 26]. The κ:λ ratio was defined as abnormal if < 0.5 or > 3 [27]. A minimum of 200 events in the plasma cell gate and 500 events in CD34+ gate on the CD34/SSC plot were collected for each tube; in order to reach this result, a total of 200,000–2,000,000 events were acquired. For each marker, an internal negative population present within the sample was used to define gates and sample fluorescence background [28, 29]. Results for total PCs and CD34+ HPCs are expressed as percentage of cells out of total acquired cells. Data for cPCs and nPCs are indicated as percentage of cells out of total PCs. Fractions of CD34 + CD19- and CD34 + CD19+ cells are reported as percentage of cells out of total CD34+ HPCs. CD34 + CD19+ Pro B cells were identified through the available markers accordingly to their recognized immunophenotype [30].

### Statistical analysis

Continuous non normal data are expressed as median values (range); normal variables are indicated as mean ± SD. Baseline differences between groups were assessed by the chi-square test or Fisher’s exact test, as needed for categorical variables (with Bonferroni correction for multiple comparisons). The univariate analysis of variance (ANOVA) was performed for parametric variables, and post hoc analysis with the Tukey’s test was used to determine pairwise differences. The Mann-Whitney *U*-test was used for intragroup analysis. The Kruskal-Wallis statistic test with pairwise comparisons was performed for nonparametric analysis. Spearman’s correlation analysis was performed to assess the statistical association between variables. Data were analyzed by IBM SPSS Software 22 version (IBM Corp., Armonk, NY, USA). All *p*-values were two-sided and *p* < 0.05 was considered statistically significant. Graphics were interpreted and presented according to published conventions [31].

## Additional file

Additional file 1: Table S1. Characteristics of the instrument. **Table S2**: Antibodies used in this study.
