# Supplementary material for: Correlation between CD117+ myeloma plasma cells and hematopoietic progenitor cells in different categories of patients
Source: Immun Ageing. 2015 Jun 4;12:5. doi: 10.1186/s12979-015-0032-1 (PMC4476172; doi:10.1186/s12979-015-0032-1)
Supplement: Additional file 1: Table S1. — Characteristics of the instrument. Table S2: Antibodies used in this study. [file 12979_2015_32_MOESM1_ESM.docx]

**Additional file 1**

| **Additional Table 1. Characteristics of the instrument.** | | |
| --- | --- | --- |
| **Model (manufacturer)** | **Lasers** | **Detector Filters** |
| Navios (Beckman Coulter) | Blue Solid State Diode: 488nm, 22mW laser output | Forward Scatter: 488/10 |
| 10 colors, 3 lasers (5+3+2 configuration) |  | Blue Laser: 525/40, 575/30, 620/30, 675/20, 695/30, 755LP |
|  |  |  |
|  | Red Solid State Diode: 638nm, 25mW laser output | Red Laser: 660/20, 725/20, 755 LP |
|  |  |  |
|  | Violet Solid State Diode: 405nm, 40mW laser output | Violet Laser: 450/50, 550/40 |
|  |  |  |
| Beckman Coulter (Miami, FL, USA) | | |

| **Additional Table 2.** Antibodies used in this study | | | | | |
| --- | --- | --- | --- | --- | --- |
| **Antigen** | **Manufacturer** | **Catalogue #** | **Clone** | **Fluorochrome** | **Isotype** |
| CD19 | Beckman Coulter | A86355 | J3-119 | PB | IgG1 Mouse |
| CD27 | Beckman Coulter | B12701 | 1A4CD27 | APCA750 | IgG1 Mouse |
| CD27 | BD Biosciences | 340424 | L128 | FITC | IgG1 Mouse |
| CD34 | Beckman Coulter | A89309 | 581 | APCA750 | IgG1 Mouse |
| CD38 | Beckman Coulter | A70205 | LS198-4-3 | PC5.5 | IgG1 Mouse |
| CD45 | Beckman Coulter | A96416 | J.33 | KO | IgG1 Mouse |
| CD56 | Beckman Coulter | IM2073U | N901 (NKH-1) | PE | IgG1 Mouse |
| CD56 | Beckman Coulter | A51078 | N901 (NKH-1) | PC7 | IgG1 Mouse |
| CD117 | Beckman Coulter | IM3698 | 104D2D1 | PC7 | IgG1 Mouse |
| CD138 | Beckman Coulter | A87787 | B-A38 | APC | IgG1 Mouse |
| κ CHAIN | Beckman Coulter | A64828 | Polyclonal | FITC | F(ab’)_2_ Rabbit |
| λ CHAIN | Beckman Coulter | A64827 | Polyclonal | PE | F(ab’)_2_ Rabbit |
| Catalogue #, catalogue number; PB, Pacific Blue; APCA750, Allophycocyanin-Alexa Fluor 750; FITC, Fluorescein Isothiocyanate; PC5.5, R-Phycoerythrin-Cyanin 5.5; KO, Krome Orange; PE, R-Phycoerythrin; PC7, R-Phycoerythrin-Cyanin 7; APC, Allophycocyanin. Beckman Coulter (Miami, FL, USA), BD Biosciences (San Jose, CA, USA) | | | | | |
